# Alterations in intramuscular connective tissue in hypertonic muscle: a scoping review

**DOI:** 10.3389/fphys.2025.1720927

**Published:** 2026-01-07

**Authors:** Xiaoxiao Zhao, Yunfeng Sun, Caterina Fede, Carmelo Pirri, Wei Gong, Alessandra Del Felice, Carla Stecco

**Affiliations:** 1 Institute of Human Anatomy, Department of Neurosciences, University of Padova, Padova, Italy; 2 Padova Neuroscience Center, University of Padova, Padova, Italy; 3 Rehabilitation medicine Department, Dongguan Kanghua Hospital, Dongguan, Guangdong, China; 4 Section of Neurology, Department of Neuroscience, University of Padova, Padova, Italy

**Keywords:** hypertonia, intramuscular connective tissue, fascia, extracellular matrix, collagen

## Abstract

**Introduction:**

Muscle hypertonia is a common symptom in patients with upper motor neuron disorders. To date, the role of intramuscular connective tissue (IMCT) alterations in hypertonic muscle has not been fully explored. This review aimed to identify and characterize alterations in IMCT components in hypertonic muscle in central neurological disorders.

**Methods:**

This scoping review included studies investigating IMCT alterations in hypertonic muscles resulting from central neurological disorders. Four electronic databases, including PubMed/Medline, CINAHL, Web of Science, and Scopus, were searched to identify relevant studies published prior to 20 July 2025. The review followed the Systematic Reviews and Meta-Analyses Extension for Scoping Reviews (PRISMA-ScR) checklist. The risk of bias was evaluated using ROBINS-E. Data were extracted and narratively synthesized according to IMCT categories.

**Results:**

Twelve studies were included. Among the included studies, increased collagen, glycosaminoglycan content, fascia thickness, and fibroblasts, as well as altered IMCT structural properties, were found in hypertonic muscles. The collagen content was found to be positively correlated with spasticity and stiffness. A key limitation of these studies is that all participants were in the chronic stage of the neurological disease.

**Conclusion:**

This scoping review provides evidence that alterations in IMCT components in muscle with hypertonia occur across different neurological conditions. Targeting these changes may provide a new intervention strategy to reduce muscle stiffness and improve the muscle function of patients with hypertonia secondary to neurological disease.

## 1 Introduction

Muscle hypertonia is a common clinical sign in patients with upper motor neuron disorders and is defined as an abnormally increased resistance to externally imposed passive muscle stretch (Ganguly et al., 2021; Evans et al., 2017), which impairs patients’ motor functions and daily activities (Ganguly et al., 2021). Within the syndrome of spastic paresis after upper motor neuron lesions, this abnormally increased resistance reflects both neural and non-neural mechanisms (Gracies, 2005a; Gracies, 2005b; Baude et al., 2019; Lorentzen et al., 2018; Gracies et al., 2025). Neural contributions include stretch hyperreflexia (spasticity), spastic dystonia and abnormal co-contraction, whereas non-neural contributions include altered mechanical properties of muscle and surrounding tissues, such as reduced elasticity, increased viscosity and structural shortening (contracture) (Evans et al., 2017; Gracies, 2005a; Baude et al., 2019). In this framework, spasticity refers specifically to an enhancement of velocity-dependent stretch reflexes measured at rest, whereas spastic dystonia denotes tonic, involuntary muscle activation at rest that contributes, together with muscle and soft-tissue alterations, to clinically observed hypertonia (Gracies, 2005b; Baude et al., 2019; Lorentzen et al., 2018). Consistent with the recent European consensus, this clinical phenomenon corresponds to what has been termed “hyper-resistance”, emphasising that the increased resistance encountered during passive stretch may arise from both neural and non-neural mechanisms (van den Noort et al., 2017). Recent evidence suggests that non-neural elements, including changes in muscle fiber and connective tissue (i.e., intramuscular connective tissue (IMCT) and the extracellular matrix (ECM)) and their mechanical properties, play a crucial role in the development of hypertonia, particularly in chronic stages of neurological diseases (Lieber et al., 2004; Dietz and Sinkjaer, 2007; Handsfield et al., 2022; Yucesoy and Huijing, 2007; Raghavan, 2018; Burke et al., 2013; Stecco et al., 2014).

Changes in muscle fiber type, length, and size have been widely studied across various central neurological disorders with hypertonia as a clinical sign, including cerebral palsy (CP), stroke, multiple sclerosis, and Parkinson’s disease (Noguchi et al., 2023; Dalise et al., 2020; Tisha et al., 2019). Research on IMCT alterations in hypertonic muscle remains relatively limited, and the contribution of IMCT to hypertonia is not yet well understood. In the muscle, IMCT refers to epimysium, perimysium, and the endomysium, which are composed of cells and the ECM (Purslow, 2020). The cellular components of the IMCT include telocytes, immune cells, myofibroblasts, and fibroblasts (Contreras et al., 2021). The main constituents of the ECM are protein fibers (such as collagen and elastic fibers) and ground substance (proteoglycans, glycosaminoglycans, and water) (Gillies and Lieber, 2011). In addition, the epimysium, perimysium, and endomysium are considered types of fascia (Stecco et al., 2025). Existing studies indicate that increased collagen deposition within the perimysium contributes to muscle stiffness in hypertonic muscle (de Bruin et al., 2014). Additionally, since the ECM plays a crucial role in force transmission and passive muscle mechanics (Gillies and Lieber, 2011), ECM remodeling could further exacerbate hypertonia by altering passive muscle mechanics (Smith et al., 2011).

To date, the role of IMCT alterations in hypertonic muscle has not been fully explored. Therefore, this review aims to systematically map the literature on IMCT alterations in muscle hypertonia in central neurological diseases by synthesizing current findings and identifying current gaps in the knowledge. Additionally, it seeks to highlight future research directions in rehabilitation science and provide evidence-based treatment recommendations for clinical practice.

## 2 Materials and methods

### 2.1 Study design

This review was conducted following the framework outlined by Levac et al. (2010) (Levac et al., 2010) and adhered to the Preferred Reporting Items for Systematic Reviews and Meta-Analyses Extension for Scoping Reviews (PRISMA-ScR) checklist (Tricco et al., 2018) (Tricco et al., 2018). The protocol of this review was registered with the International Prospective Register of Systematic Reviews database (PROSPERO: CRD420251018368).

### 2.2 Research questions

This review aimed to identify the alterations in IMCT components in hypertonic muscle in central neurological disorders.

### 2.3 Eligibility criteria

The inclusion criteria were as follows: 1) studies involving human or animal models exhibiting muscle hypertonia; 2) studies reporting structural, biochemical, and histological alterations of the IMCT in hypertonic muscles (e.g., fascia, intramuscular connective tissue, cells, telocytes, immune cells, myofibroblasts, fibroblasts ECM, collagen, hyaluronan, elastic fiber); and 3) full-length, peer-reviewed, original research articles written in English, published before 20 July 2025 since inception.

The exclusion criteria were as follows: 1) studies focusing solely on neurogenic causes of muscle hypertonia; and 2) conference abstracts, editorials, or reviews without primary data (further details on the eligibility criteria are provided in Supplementary Material S1).

### 2.4 Data sources and search strategy

A systematic search was independently conducted by two reviewers (XZ and YS) using the following electronic databases: PubMed/Medline, CINAHL, Web of Science, and Scopus. The search terms included: ((fascia) OR (intramuscular connective tissue) OR (extracellular matrix) OR (elastic fiber) OR (elastic fiber) OR (collagen) OR (hyaluron*) OR (telocytes) OR (immune cells) OR (myofibroblasts) OR (fibroblasts)) AND ((muscle hypertonia) OR (muscle spasticity) OR (muscle spastic dystonia) OR (muscle rigidity) OR (muscle hyperreflexia) OR (increased pyramidal tone)). The electronic database search was supplemented by screening the reference lists of included studies and relevant reviews. Results were exported to the EndNote bibliographic software, and duplicates were removed. Details on the development and rationale for the search terms are provided in Supplementary Material S2, and the full search strategy is presented in Supplementary Material S3–S6.

### 2.5 Study selection

The study selection process was performed in two stages. First, after removing duplicate articles, all titles and abstracts identified through the search were screened independently by two researchers (XZ and YS) to exclude studies that did not meet the eligibility criteria. In the second stage, the full-text articles of the remaining studies were reviewed independently by the same researchers (XZ and YS) to confirm eligibility. Any disagreements between researchers were resolved through discussion with a third researcher (CS). Inter-rater reliability (the kappa statistic) was applied to check the agreement between the two independent researchers (XZ and YS). The categories of the agreement were defined as follows: fair agreement, Kappa = 0.40–0.59; good agreement, Kappa = 0.60–0.74; excellent agreement, Kappa ≥0.75 (Bowling and Ebrahim, 2005).

### 2.6 Risk of bias appraisal

The risk of bias in non-randomized studies of Exposure (ROBINS-E) assessment tool (Higgins et al., 2024) was used to evaluate the methodological quality of the included studies. The appraisal was conducted by one researcher (XZ) and cross-checked by another (SY) in Excel.

### 2.7 Data extraction

A standardized data extraction form was developed in accordance with the Cochrane handbook for Systematic Reviews. This form was designed to include the key information from each study. The following data were extracted: 1) study characteristics: author(s), year of publication, study design, country of origin, and sample size; 2) population: age and sex of the participants, neurological condition, and tested muscles; 3) intervention method: non-invasive (imaging) or invasive (biochemical, histological); 4) outcomes: structural, biochemical, or historical alterations of the IMCT in muscle with hypertonia due to neurological disease; and 5) key findings: the main results and conclusions relevant to IMCT components in hypertonic muscle. The data was extracted by one researcher (XZ) and cross-checked by another (SY). Any discrepancies were resolved through discussion or consultation with a third reviewer (CS). No automation tools were used.

### 2.8 Data synthesis

Data from the included studies were narratively synthesized according to the IMCT components and the evaluation method used.

## 3 Results

### 3.1 Study selection

**FIGURE 1:**
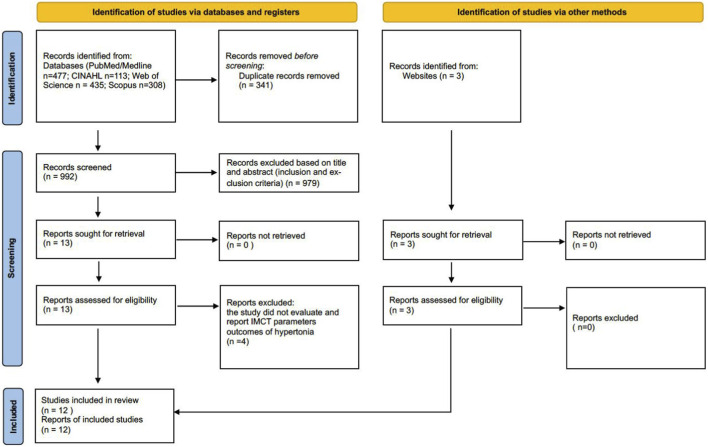
PRISMA flow chart for study selection.

The systematic search retrieved 1,333 records. Following the removal of 341 duplicates, 992 articles were excluded at the title and abstract screening stage according to the inclusion and exclusion criteria (Supplementary Material S1). Three articles were retrieved using a manual search. In total, 16 articles underwent full-text assessment, of which four studies were excluded, leaving 12 studies included in the final analysis (Figure 1). The inter-rater agreement was excellent between the two independent reviewers (Kappa = 0.846). The following details are presented in the Supplementary Material: search results (S3–S6); a list of full-text articles with the independent decision of the researchers (S7); the inter-rater reliability calculation (S8); and a list of full-text articles excluded, along with the reasons for exclusion (S9).

### 3.2 Study characteristics

The characteristics of the 12 included studies are summarized in Table 1. All studies report data on human participants. Eleven were cross-sectional, while one was experimental. The conditions investigated included CP (nine studies) (de Bruin et al., 2014; Smith et al., 2011; Booth et al., 2001; Malaiya et al., 2007; Gagliano et al., 2013; Gagliano et al., 2009; Smith et al., 2021; Von Walden et al., 2018; Wohlgemuth et al., 2024), stroke (two studies) (Menon et al., 2019; Choi et al., 2024), spinal cord injury (SCI) (one study) (Olsson et al., 2006), and acquired brain injury (ABI) (one study) (Von Walden et al., 2018). The sample size in each study ranged from 9 to 88, with a nominal total of 411 participants. When accounting for the 26 participants from (Smith et al., 2021) who were also included in the 52 participants reported in (Smith et al., 2021), the actual total number of participants was 385.

**TABLE 1:** Characteristics of the 12 included studies.

No.	Study	Country	Subjects	Study design	Condition	Sample size	Gender M/F	Age mean ± SD (range) years	Muscle
1	Booth et al. (2001)	United Kingdom	Human	Cross-sectional	CP	28 CP:26 Ctr:2	CP: 14/12 Diplegia: 5/8 Quadriplegia: 9/4 Ctr: 2/0	CP: 10.6 ± 0.6 (4–17) Ctr: 10.33 and16.23	LL: vastus lateralis muscle
2	Malaiya et al. (2007)	United Kingdom	Human	Cross-sectional	CP	31 CP:16 Ctr:15	CP: 4/12 Ctr: 6/9	CP: 7.8 (4–12) Ctr: 9.5 (4–13)	LL Medial Gastrocnemius
3	Gagliano et al. (2009)	Italy	Human	Cross-sectional	CP	28 CP: 21 Ctr: 7	CP: 10/11 (Diplegia: 3/3 Quadriplegia: 7/8) Ctr: 5/2	CP Diplegia: 15.33 ± 2.42 Quadriplegia:13.67 ± 3.6 Ctr: 17.29 ± 1.89	LL Gracilis and semitendinosus tendon
4	Smith et al. (2011)	United States of America	Human	Cross-sectional	CP	52 CP:33 Ctr:19	CP: 23/10 Ctr: 8/11	CP: 9.6 ± 4.2 Ctr: 15.8 ± 1.8	LL Gracilis and semitendinosus muscles
5	de Bruin et al. (2014)	Netherlands	Human	Cross-sectional	CP	39 CP: 29 Ctr:10	CP: 15/14 Ctr: 3/7	CP:19 (5–40) Ctr: 45 (21–62)	UL Flexor carpi ulnaris (FCU) muscle
6	Smith et al. (2021)	UAS	Human	Cross-sectional	CP	26 CP:13 Ctr:13	Not reported	CP: 10.4 ± 4.4 Ctr: 15.5 ± 2.0	LL: semitendinosus
7	Gagliano et al. (2013)	Italy	Human	Cross-sectional	CP	9 CP:4 Ctr:5	Not reported	CP: 11,5 ± 5,5 Ctr: 12,6 ± 3,7	LL: tendon of Gracilis, Semitendinosus muscles
8	Menon et al. (2019)	United States of America	Human	Experimental	Stroke	10 Stroke:5 Ctr:5	Stroke: 2/3 Ctr: 1/4	Stroke: 52 ± 5 Ctr: 27 ± 2	UL Biceps brachii, triceps brachii
9	Choi et al. (2024)	Korea	Human	Cross-sectional	Stroke	88 CP:88 Ctr: contralateral	stroke: 56/32	CP: 62.7 ± 13.0	LL: tibialis anterior, peroneus longus, gastrocnemius, and soleus muscles
10	Olsson et al. (2006)	Sweden Germany	Human	Cross-sectional	SCI	14 SCI: 7 Ctr:7	SCI: 7/0 Ctr: 7/0	SCI: 44 ± 2 (38–51) Ctr: 44 ± 2 (37–52)	LL Vastus Lateralis Muscle
11	Wohlgemuth et al. (2024)	United States of America	Human	Cross-sectional	CP	56 CP:41(CPH:27; CPA:14) Ctr:15	CP: 26/15 (CPH:17/10; CPA:9/5) Ctr: 6/9	CP: CPH: 10.52 ± 3.04 CPA: 9.36 ± 4.41 Ctr: 14.72 ± 1.87	LL Gracilis muscle (n = 27); adductor longus muscle (n = 14)
12	Von Walden et al. (2018)	Sweden	Human	Cross-sectional	CP/ABI	30 CP/ABI:20 Ctr:10	CP/ABI:17/3 Ctr8/2	CP/ABI: 15.5 (9–18); Ctr: 15.1 (7–21)	UL Biceps brachii

The muscle sampling sites varied across studies. Three studies focused on the upper limbs, including the flexor carpi ulnaris (de Bruin et al., 2014), the biceps brachii (Von Walden et al., 2018; Menon et al., 2019), and the triceps brachii (Menon et al., 2019). The remaining nine studies focused on the lower limbs, with muscle sampling sites including the vastus lateralis (Booth et al., 2001; Olsson et al., 2006), the gastrocnemius (Malaiya et al., 2007; Choi et al., 2024), the gracilis (Smith et al., 2011; Gagliano et al., 2013; Gagliano et al., 2009; Wohlgemuth et al., 2024), the semitendinosus (Smith et al., 2011; Gagliano et al., 2013; Gagliano et al., 2009), the adductor longus muscle (Wohlgemuth et al., 2024), the tibialis anterior, the peroneus longus, and the soleus muscle (Choi et al., 2024).

The characteristics of the study design of the 12 included studies are summarized in Table 2. In all studies, participant inclusion was based on a medical diagnosis. Ten studies reported the level of hypertonia (de Bruin et al., 2014; Smith et al., 2011; Booth et al., 2001; Malaiya et al., 2007; Gagliano et al., 2009; Von Walden et al., 2018; Wohlgemuth et al., 2024; Menon et al., 2019; Choi et al., 2024; Olsson et al., 2006); however, two did not provide this information (Gagliano et al., 2013; Smith et al., 2021). Properties of the IMCT were evaluated in nine studies through invasive muscle biopsy (de Bruin et al., 2014; Smith et al., 2011; Booth et al., 2001; Gagliano et al., 2013; Gagliano et al., 2009; Smith et al., 2021; Von Walden et al., 2018; Wohlgemuth et al., 2024; Olsson et al., 2006). From the remaining three studies, one assessed the IMCT properties using non-invasive imaging (Malaiya et al., 2007; Menon et al., 2019; Choi et al., 2024).

**TABLE 2:** Characteristics of the study design.

Studies	Patient inclusion criteria	Level of hypertonia	Evaluation technique			Comparison method	
			Non-invasive	Invasive		Between limb	Between subjects
			Imaging	Biochemical	Histological		
Booth et al. (2001)	CP: diplegic and quadriplegic surgery patient without neuromuscular disorders other than CP Ctr: normally developing children undergoing orthopedic surgery	MAS: mild (n = 5); moderate (n = 9); mod-severe (n = 9); severe (n = 3)		X			X
Malaiya et al. (2007)	CP: children with spastic hemiplegic cerebral palsy (SHCP) with limited passive dorsiflexion range had no previous surgical intervention and had not received Botulinum toxin in the 6 months prior to scanning Ctr: typically developing children	Limited passive dorsiflexion range was used as an indicator of muscle hypertonia	X				X
Gagliano et al. (2009)	CP: CP patients for tendon lengthening procedure Ctr: ACL surgery patient	Diplegic: lower limb Ashworth 2–3 (n = 6) Quadriplegic: lower limb Ashworth 4–5 (n = 15)		X			X
Smith et al. (2011)	CP: children with CP undergoing hamstring lengthening surgery Ctr: age matched ACL surgery patient	popliteal angle: 114 ± 15		X	X		X
Gagliano et al. (2013)	CP: CP persons undergoing tendon lengthening procedure Ctr: ACL surgery patient	Not reported			X		X
de Bruin et al. (2014)	CP: patients undergoing upper extremity surgery with CP and a Zancolli type IIa or IIb grasp and release pattern Ctr: required upper limb surgery patient	Zancolli type IIa or IIb grasp and release pattern			X		X
Smith et al. (2021)	CP: CP children for tendon lengthening procedures Ctr: ACL surgery patient	Not reported		X			X
Menon et al. (2019)	Stroke: chronic stroke patient with upper limb muscle stiffness Ctr: health volunteer	Moderate to severe muscle stiffness	X				X
Choi et al. (2024)	Stroke: chronic hemiplegia (onset > 6 month) and MAS ≥2 in the lower limbs Ctr: contralateral side	MAS = 2 (n = 59) MAS = 3 (n = 29)	X			X	
Olsson et al. (2006)	SCI: spinal cord-injured patients with spasticity Ctr: age- and sex-matched control subjects with no history of neuromuscular disease	MAS:1–4			X		X
Wohlgemuth et al. (2024)	CP: children with spastic undergoing hamstring or adductor lengthening surgery Ctr: children undergoing ACL reconstruction with hamstring autograft	CPH popliteal angle: 89 ± 15 CPA:N/A		X	X		X
Von Walden et al. (2018)	CP/ABI: children and adolescents with spasticity for biceps tendon lengthening surgery Ctr: postmortem healthy donors (7–21 years)	Extension deficit >10° at the elbow		X	X		X

The majority of the studies (11/12) compared IMCT alterations in hypertonic muscle to healthy controls (de Bruin et al., 2014; Smith et al., 2011; Booth et al., 2001; Malaiya et al., 2007; Gagliano et al., 2013; Gagliano et al., 2009; Smith et al., 2021; Von Walden et al., 2018; Wohlgemuth et al., 2024; Menon et al., 2019; Olsson et al., 2006), while one study compared IMCT alterations to the contralateral side in the same participant (Choi et al., 2024). Eight studies recruited surgical children with CP as the experimental group, with typically developing children undergoing orthopedic surgery as the control group. One recruited surgical children with CP/ABI with postmortem health donors as a control group. The study on SCI-induced hypertonia recruited patients with SCI with spasticity, comparing IMCT alterations with age-and sex-matched control subjects (Olsson et al., 2006). Both stroke-induced hypertonia studies recruited patients with stroke with hypertonia in the chronic stroke stage, investigating the IMCT properties by non-invasive imaging. One compared patients with stroke with healthy controls (Menon et al., 2019), while the other compared with the contralateral side (Choi et al., 2024).

Three studies reported thickness and structural alterations of the IMCT of hypertonic muscles (de Bruin et al., 2014; Malaiya et al., 2007; Choi et al., 2024), nine studies focused on ECM alterations in the IMCT of hypertonic muscle (Smith et al., 2011; Booth et al., 2001; Gagliano et al., 2013; Gagliano et al., 2009; Smith et al., 2021; Von Walden et al., 2018; Wohlgemuth et al., 2024; Menon et al., 2019; Olsson et al., 2006), two studies evaluated the mRNA level of collagen (Gagliano et al., 2009; Von Walden et al., 2018), and one study investigated fibroblast cells in the IMCT of hypertonic muscle (Smith et al., 2021).

### 3.3 Changes to the IMCT thickness and structure

Fascia thickness was assessed in two studies using different methodologies (Table 3). One study measured fascia thickness in CP-induced hypertonic muscles through histological analysis (using Sirius red staining to identify collagen fiber) of a biopsy (de Bruin et al., 2014), while the other employed an indirect ultrasound-based method to assess fascia thickness in stroke-induced hypertonic muscles (Choi et al., 2024). Despite the methodological differences, both studies reported increased fascia thickness in hypertonic muscles. Specifically, in individuals with CP, the tertiary perimysium of the Flexor carpi ulnaris (FCU) was found to be 3-fold thicker than that in healthy controls (de Bruin et al., 2014). In patients with chronic stroke, the fascia thickness increased 1.32 to 1.35-fold in the crural fascia and epimysial fascia of the tibialis anterior muscle on the affected spastic side compared to the unaffected side (Choi et al., 2024). In addition, IMCT structural alterations were evaluated using the Deep Fascicle Aponeurosis (DFA) angle in one study (n = 31) using 3D ultrasound, which showed that the DFA was approximately 2° shallower in children with CP than that in typically developing children (Malaiya et al., 2007).

**TABLE 3:** Methods and main findings of the studies.

Studies	Methods	Main findings
Non-invasive imaging		
Menon et al. (2019)	MRI: glycosaminoglycan (GAG) content	GAG content increased 1.31-fold in the biceps in stroke patients, which reduced after hyaluronidase injection
Choi et al. (2024)	Ultrasound: fascia thickness	Fascia thickness increased 1.32 to 1.35-fold in the crural fascia and epimysial fascia of the tibialis anterior muscle
Malaiya et al. (2007)	3D Ultrasound: the deep fascicle aponeurosis angle (DFA)	DFA was 2^0^ shallower in both lower limbs of CP children compared to typically developing children
Biochemical		
Booth et al. (2001)	Hydroxyproline assay (colorimetric): collagen content	Collagen content increased 2.5-fold in CP children
Gagliano et al. (2009)	Real-time PCR: mRNA of ECM components - collagen type I (*COL1A2*) - enzyme responsible for collagen maturation lysyl hydroxylase 2b (*LH2b*) - matrix metalloproteinase-1 (*MMP-1*) - tissue inhibitor of MMP (*TIMP-1*) - transforming growth factor-β1 (*TGF-β1*), and - secreted protein acidic and rich in cysteine (*SPARC*)	- *COL1A2, LH2b,* and *SPARC* mRNA levels were significantly higher expressed in CP tendons - *MMP-1* and *TIMP-1* were expressed in normal tendons with high interindividual variability - A trend toward upregulation of *TGF-β1* mRNA levels in CP tendons, but without statistical significance - Hypertonic quadriplegic subjects displayed higher mRNA levels of *COL1A2, LH2b, TGF-β1,* and *SPARC* than the diplegic group
Smith et al. (2011)	Hydroxyproline assay (colorimetric): collagen content	Collagen content was increased 1.4-fold in the gracilis muscle and 2.2-fold in the semitendinosus muscle in CP
Smith et al. (2021)	ELISA - Collagen content (types I, III, IV, VI) - Proteoglycans (decorin and biglycan) - Glycosaminoglycans (Uronic acid and HA) Hydroxyproline assay (HPLC): collagen content Fluorescence-based HPLC following acid hydrolysis: collagen cross-links (hydroxylysyl pyridinoline (HP), lysyl pyridinoline (LP), and pentosidine)	- Collagen content (types I, III, IV, VI) and total collagen increased 3-5-fold in CP muscle - GAG: uronic acid 2-fold increase in CP. HA not significantly altered in CP. - Overall proteoglycan unchanged, decorin increased 2.7-fold, biglycan decreased 2.7-fold in CP. - Collagen cross-links: hydroxylysyl pyridinoline (HP) and lysyl pyridinoline (LP) were significantly elevated (2–3 fold) in CP muscle; pentosidine was not significantly changed in CP.
Wohlgemuth et al. (2024)	Hydroxyproline assay (colorimetric): collagen content Collagen solubility assay: collagen cross-links	No significant difference in collagen content and collagen cross-links between CP and control group
Von Walden et al. (2018)	Real-time PCR: mRNA of ECM components - collagen type I (*COL1A1*) - connective tissue growth factor (*CTGF*) - transforming growth factor-β1 (*TGF- β1*) - transforming growth factor beta receptor 2 (*TGFBR2*) - latent transforming growth factor beta binding protein 1 (*LTBP1*), and - lysyl oxidase (*LOX*)	mRNA expression of ECM-related genes was significantly upregulated in CP/ABI muscle compared to controls: *COL1A1* (2.1-fold), *CTGF* (3.4-fold), *TGF-β1* (1.7-fold), *TGFBR2* (1.6-fold), *LTBP1* (1.5-fold), and *LOX* (1.9-fold)
Histological		
Smith et al. (2021)	Stereological analysis and imaging - volume fractions of the ECM - cellular components in IMCT	- A significant increase in collagen fibrils organized in cables - An increased volume fraction of fibroblasts in CP muscle
Smith et al. (2011)	Immunohistochemistry - Col I - Laminin	Col I and laminin increased in CP muscle
Booth et al. (2001)	Immunohistochemistry staining (qualitative analysis by observation): Col I	- Collagen I increased in the endomysium in spastic muscle - Increased inter-fiber space in spastic muscle associated with excessive connective tissue
de Bruin et al. (2014)	Picro-Sirius Red staining (quantitative analysis by ImageJ software): collagen	Primary and secondary perimysium thickness were unchanged, but tertiary perimysium was 3-fold thicker in CP muscle. This thickening was observed in most spastic muscles but not in all cases, indicating some variability
Gagliano et al. (2013)	Picro-Sirius Red staining (semiquantitative analysis by five-point scoring system): collagen Alcian Blue (semiquantitative Analysis by five-point scoring system): GAG	- Collagen staining intensity increased around 3-fold in the CP gracilis tendon, and slightly increased in the semitendinosus tendon - GAG staining intensity increased 2.7-fold in the CP gracilis tendon, and slightly increased in the semitendinosus tendon
Olsson et al. (2006)	Toluidine Blue staining (qualitative analysis by observation): collagen amount	Collagen accumulation and connective tissue expansion in SCI-induced hypertonia muscle
Wohlgemuth et al. (2024)	Second Harmonic Generation (SHG): collagen alignment	- No significant difference in collagen alignment index at matched sarcomere length between CP and typically developing children - Alignment increased significantly with strain in all groups. CPA muscles showed reduced collagen realignment under strain
Von Walden et al. (2018)	Picro-Sirius Red staining (quantitative analysis by Leica Qwin Pro software): collagen	Collagen content increased 31% (1.31-fold) in the perimysium in the CP/ABI muscle

### 3.4 Changes to the ECM components

The collagen content was investigated in 8/12 studies (Table 3). Among these, three studies (Smith et al., 2011; Booth et al., 2001; Smith et al., 2021) used both biochemical and histological assessments, and one study (Wohlgemuth et al., 2024) applied a biochemical method to evaluate collagen levels in CP-induced hypertonic muscle. Four studies applied histology methods, including one in SCI-induced hypertonic muscle (Olsson et al., 2006), one in CP/ABI-induced hypertonic muscle (Von Walden et al., 2018), and two in the muscles or tendons of individuals with CP (de Bruin et al., 2014; Gagliano et al., 2013). Biochemical quantification (Smith et al., 2011; Booth et al., 2001; Smith et al., 2021) and histological semiquantitative analysis (Gagliano et al., 2013) reported a 1.4-fold–5-fold increase in total collagen in CP-induced hypertonic muscles. In the study on CP/ABI-induced hypertonic muscle, histological quantitative analysis showed that the collagen content increased by 31% in the perimysium (Von Walden et al., 2018). Furthermore, histological qualitative analysis showed collagen accumulation in SCI-induced hypertonic muscle (Olsson et al., 2006), as well as in CP-induced hypertonic muscles (de Bruin et al., 2014; Smith et al., 2011; Booth et al., 2001; Smith et al., 2021), based on histological morphological observations. In contrast, Wohlgemuth et al. found no significant difference in the collagen content between patients with CP and controls. Regarding the collagen subtypes, 3-fold–5-fold increases in collagen type I, III, IV, and VI were reported in CP-induced hypertonic muscles, as assessed using biochemical methods (Smith et al., 2021). In addition, the histological analysis showed increased collagen type I in the lower limb muscles of patients with CP using immunohistochemistry (Smith et al., 2011; Booth et al., 2001).

In addition to the collagen amount, two studies investigated the structural organization of collagen in CP-induced hypertonic muscle using both biochemical and histological methods (Smith et al., 2021; Wohlgemuth et al., 2024). Smith et al. demonstrated that collagen cross-links with hydroxylysyl pyridinoline (HP) and lysyl pyridinoline (LP) were elevated 2–3-fold in CP muscle, as assessed with fluorescence-based HPLC following acid hydrolysis, along with a significant increase in collagen fibrils organized in cables, as assessed by stereological analysis. In contrast, Wohlgemuth et al. found no significant difference in collagen cross-links between patients with CP and controls using the hydroxyproline assay and collagen solubility assay. They further used Second Harmonic Generation (SHG) imaging to assess collagen fiber alignment. The collagen alignment index did not differ significantly between CP and control muscles at a matched sarcomere length. Furthermore, it increased significantly with strain in all groups, and CP muscles showed reduced collagen realignment under strain.

Two studies evaluated the mRNA level of collagen type I (*COL1A1*) (Von Walden et al., 2018) and *COL1A2* (Gagliano et al., 2009). In CP/ABI-induced upper limb hypertonic muscles, the mRNA expression of *COL1A1* was significantly upregulated by 2.1-fold compared to typically developing controls. Similarly, in the lower limb tendons of CP-induced hypertonic muscles, the mRNA expression of *COL1A2* was significantly higher, especially higher in quadriplegic tendons, with a 25-fold increase compared to normal tendons. However, no study has investigated the collagen alterations in stroke-induced hypertonic muscles.

Glycosaminoglycan (GAG) was investigated in 3/12 studies, each using a different evaluation method (Table 3). All three studies reported an increased GAG level in hypertonic muscles or tendons. One investigated the GAG level using magnetic resonance imaging (MRI) in patients with stroke-induced hypertonia (Menon et al., 2019). Another study assessed the GAG level by histological Alcian blue staining in CP lower limb tendons (Gagliano et al., 2013). The third study assessed the GAG level in CP-induced hypertonic muscle using an ELISA (Smith et al., 2021). In the same study (Smith et al., 2021), GAG subtypes were also investigated: uronic acid levels were found to double in CP-induced hypertonic muscles, while hyaluronic acid (HA) levels were not significantly altered.

Other ECM components, such as proteoglycans, including decorin and biglycan, were evaluated using biochemical analyses (Smith et al., 2021) (Table 3). Laminin was studied using histological analysis (Smith et al., 2011). However, no studies have investigated elastic fiber, which are an important component of the ECM regarding tissue mechanics. The overall proteoglycan level remained unchanged; however, this was attributed to opposing changes in specific proteoglycans, with decorin increasing 2.7-fold and biglycan decreasing by the same magnitude (Smith et al., 2021). Furthermore, the histological study demonstrated an increase in laminin in CP-induced hypertonic muscles (Smith et al., 2011).

The gene expression levels of other ECM components were also evaluated in the lower limb tendon of CP-induced hypertonic muscle (Gagliano et al., 2009) and in the upper limb muscle of CP/ABI-induced hypertonic muscles (Von Walden et al., 2018). These genes included matrix metalloproteinase-1 (*MMP-1*) and its inhibitor tissue inhibitor of MMP-1 (*TIMP-1*); the collagen maturation enzyme lysyl hydroxylase 2b (*LH2b*); the matricellular protein secreted protein acidic and rich in cysteine (*SPARC*), which is involved in ECM remodeling; transforming growth factor-β1 (*TGF-β1*), which is a key cytokine in collagen regulation; transforming growth factor beta receptor 2 (*TGFBR2*), which mediates TGF-β signaling and regulates collagen production; latent transforming growth factor beta binding protein 1 (*LTBP1*) which modulates the bioavailability and activation of TGF-β; lysyl oxidase (*LOX*), an enzyme essential for collagen cross-linking and ECM stabilization; and connective tissue growth factor (*CTGF*), which promotes fibroblast activation and collagen synthesis in fibrotic tissue. In the lower limb tendons of patients with CP, the mRNA expression levels of *LH2b* and *SPARC* were significantly upregulated, while *TGF-β1* showed a trend toward upregulation, although it did not reach statistical significance. Furthermore, patients with CP showed higher levels of *LH2b, TGF-β1*, and *SPARC* than those with diplegic CP and healthy individuals. In the upper limb muscles of patients with CP/ABI, the mRNA expression of *CTGF, TGFB1, TGFBR2, LTBP1,* and *LOX* was significantly increased, ranging from 1.5 to 3.4-fold, compared to typically developing controls.

### 3.5 Changes of IMCT cells

In addition to ECM components, one study investigated IMCT cellular composition by stereological analysis of electron microscopy images and found that the volume fraction of fibroblasts was significantly increased in CP muscle compared with the muscle of typically developing children (Smith et al., 2021) (Table 3).

### 3.6 Correlation between ECM changes and clinical outcomes

Correlation analyses were conducted in three studies (Booth et al., 2001; Smith et al., 2021; Wohlgemuth et al., 2024) to investigate the relationship between the ECM and severity of clinical hypertonia, as well as the association between ECM components and mechanical behavior, specifically stiffness. Collagen content (evaluated by hydroxyproline assay) was found to be positively correlated with hypertonia severity, assessed by the Modified Ashworth Score (MAS), indicating that collagen levels increased with a higher MAS score (Booth et al., 2001). Similarly, (Wohlgemuth et al., 2024), reported that the total collagen content and cross-links (measured by the colorimetric hydroxyproline assay) were positively correlated with stiffness across all muscle groups. Furthermore, in the CP hamstring group, total collagen content, cross-links, and stiffness were positively correlated with the popliteal angle. Additionally, both total collagen (measured by the hydroxyproline assay) and collagen I (by ELISA), as well as the total proteoglycan level (also measured by ELISA), showed positive correlations with stiffness measured using mechanical testing of muscle fiber bundles. In contrast, biglycan (measured by ELISA) was negatively correlated with stiffness (Smith et al., 2021).

### 3.7 Risk of bias

The risk of bias was assessed using the ROBIN-E. Most domains were evaluated as low risk of bias, except for Domain 1, in which nine studies were rated as having “some concern,’ and Domain 3, in which all studies were rated as having a “high risk of bias,’ which led to all studies being rated as having a high overall risk of bias. The high risk of bias in Domain 3 (the risk of bias in the selection of participants into the study) is mainly because participants in all 12 included studies were in the chronic stage of their neurological disease (n = 12). Domain 1 (risk of bias due to confounding factors) raised some concerns (n = 9) due to a lack of information about relevant confounding factors, such as previous treatment or sex. The remaining five domains were evaluated to be low risk of bias. Domain 2 (risk of bias arising from measurement of the exposure) was evaluated to be low (n = 12) as the neurological disease-induced hypertonia in all the studies was based on clinical diagnosis, and more than half of the studies also reported the MAS result. The risk of bias due to post-exposure interventions (Domain 4) was evaluated as low (n = 12) because 11 of the 12 studies were cross-sectional studies, and the data extracted from the one experimental study for this review were collected prior to the intervention. The risk of bias due to missing data (Domain 5) was low (n = 12) as no missing data were reported in the studies. The risk of bias arising from measurement of the outcome (Domain 6) was rated as low (n = 12) since all participants, regardless of exposure group or control group, underwent the same measurement, and the knowledge of participants’ exposure history is unlikely to influence the laboratory outcomes or image evaluations. The risk of bias in the selection of the reported result (Domain 7) was rated as low (n = 12), as there was no evidence of selective reporting. All the results are presented in Supplementary Material S10.

## 4 Discussion

The studies included in this scoping review reported alterations in the ECM of the IMCT in hypertonic muscles across various conditions, primarily in children with CP, with fewer studies involving children with ABI, and adults with stroke and SCI. These changes include increased collagen accumulation and modifications in the composition of GAGs and proteoglycans, which may contribute to the severity of muscle stiffness and hypertonia.

Collagen is the most extensively studied ECM component, with the highest number of studies and largest sample sizes analyzed, primarily in pediatric populations with CP undergoing surgery. Despite differences in populations, methods, and the specific muscle examined, seven studies reported increased collagen accumulation in muscles or tendons with hypertonia. Collagen is the most abundant ECM component, playing a crucial role in structural support and resistance to mechanical forces, including tension and stretch (Ricard-Blum, 2011). Increased collagen levels are generally associated with increased fascia and muscle stiffness (Tang, 2020), which can restrict force transmission, reduce the functional capacity, and diminish the overall efficiency (Zhang et al., 2021). Correlation analyses have demonstrated that elevated collagen levels are positively associated with hypertonia severity (Booth et al., 2001) and muscle stiffness (Smith et al., 2011). The findings from genes involved in collagen turnover showed higher expression levels in quadriplegic CP compared to diplegic CP and healthy individuals, suggesting a more pronounced collagen remodeling response in severe hypertonia. Additionally, collagen subtype analyses further revealed that the levels of type I, III, IV, and VI collagen are elevated in CP-induced hypertonia, with type I being the predominant form (Smith et al., 2021). This shift suggests collagen remodeling processes that enhance muscle stiffness, which may be associated with more severe hypertonia. Furthermore, the presence of increased collagen cross-links and increased collagen fibrils organized into cable-like structures indicates that collagen alterations in CP-induced hypertonic muscle involve not only an increased amount but also structural remodeling, suggesting a potential contribution to increased tissue stiffness (Smith et al., 2021). In contrast, one study reported no significant increase in collagen content and cross-links in the CP group (Wohlgemuth et al., 2024). This finding appears to be inconsistent with the other studies, and may be partially attributable to differences in muscle types, as collagen content varies across muscles (Binder-Markey et al., 2020). Unlike most studies that assessed the same muscle type across groups (Wohlgemuth et al., 2024), evaluated the gracilis and adductor longus muscles in CP group but only the gracilis muscle in the control group. This anatomical mismatch in sampling may have introduced bias, as comparisons across different muscle types may confound the pathological differences with natural inter-muscular variability. However, seven of the eight collagen studies involved children with CP (or ABI) undergoing surgery for spasticity, while one examined adults with SCI. Therefore, the evidence is limited by population and methodological heterogeneity, which introduces limitations in generalizability and cross-study comparability.

GAG levels were reported to increase in three studies using different methods, whereas one study found no significant change in the total proteoglycan content. However, the specific subtypes of GAG components, such as HA and uronic acid, as well as other subtypes of proteoglycan components, such as decorin and biglycan, exhibited differential alterations. When combined with the correlation analysis, total proteoglycan level was identified as a positive predictor, while biglycan was identified as a negative predictor of muscle stiffness. This suggests that individual ground substance components may contribute differently to muscle stiffness in hypertonic muscle.

In addition to ECM components, Smith et al. (2021) investigated the cellular components of IMCT. Their findings demonstrated a significant increase in the volume fraction of fibroblasts in CP-induced hypertonic muscle. Fibroblasts are the predominant cell type in IMCT (Langevin et al., 2004). They are responsible for secreting precursors of ECM components (such as collagen and elastic fibers) to maintain the integrity of the IMCT (Fede et al., 2021). An increase in fibroblast density may contribute to elevated ECM production, particularly collagen, and promote fibrotic remodeling of the tissue (Moretti et al., 2022). In the context of CP-induced hypertonia, the increased volume fraction of fibroblasts might partially explain the elevated collagen content observed in the ECM and the increase in tissue stiffness.

No studies have investigated the alteration or contribution of elastic fibers in hypertonic muscles, representing a significant gap in the understanding of ECM adaptations in hypertonia. Elastic fibers are a type of protein fiber in the ECM, primarily composed of elastin and fibrillin, which provide resilience and flexibility to tissues (Kielty et al., 2002). This function is particularly essential for maintaining normal muscle mechanics, ensuring proper force transmission, and preventing excessive stiffness (Muiznieks and Keeley, 2013; Ritty et al., 2003). In hypertonic muscles, the lack of research on elastic fiber alterations leaves a critical gap in understanding the broader ECM changes in hypertonia. Given that elastic fibers interact with collagen to regulate tissue flexibility, their potential degradation or altered synthesis in hypertonic muscles may impact mechanical properties (Sherratt, 2009). Future research should investigate whether elastic fiber alterations contribute to reduced muscle compliance and whether this exacerbates hypertonia.

For the risk of bias assessment of the included studies, according to the ROBINS-E, all included studies were rated as having an overall high risk of bias. This is mainly due to the high risk of bias in the participant selection (Domain 3), as all the included studies recruited participants in the chronic stage of neurological conditions. The recruitment strategy limits the ability to evaluate the IMCT alterations across different stages of hypertonia. In addition, approximately 83% of the studies were rated as having “some concern’ in the risk of bias due to confounding factors (Domain 1) owing to a lack of reporting the confounders, such as sex and previous treatment. While the remaining five domains were rated as low risk of bias, the high risk of bias in participant selection suggests the limitations in the current evidence base and reduces the generalizability of the findings of this review.

### 4.1 Clinical implications

The findings of this review highlight the potential contribution of the IMCT and its composition to muscle hypertonia across different neurological conditions. Traditionally, focal injections, such as Botulinum toxin A (BoNT-A), have primarily targeted the neural components of hypertonia. However, evidence from this review suggests that fascial thickening and ECM alterations may also play a role in increased muscle stiffness in hypertonic muscle, particularly in chronic and severe cases. Data on stroke have shown that neural contributions decrease with severity, while non-neural contributions were predominant in severe cases (Wang et al., 2017). These findings suggest the need for broader treatment strategies to treat both the neural and structural contributors. BoNT-A alone may be insufficient as a comprehensive treatment, and additional therapies should consider ECM alterations.

Stretching interventions are commonly used strategy in clinical practice to address hypertonia. A Cochrane review reported that conventional, relatively low-dose stretching programmes generally produce only small changes in joint range, which are unlikely to be clinically important at the joint level (Harvey et al., 2017). More recently, a systematic review of chronic stretching in people with stroke and reduced joint mobility found that most short-to medium-term protocols did not meaningfully alter muscle mechanical properties or architecture, whereas a year-long, high-dose plantarflexor stretching programme increased fascicle length and ankle dorsiflexion range of motion (Lecharte et al., 2020). Although these studies did not directly assess ECM or IMCT composition, they suggest that sufficiently intensive stretching may influence muscle architecture and passive mechanics, potentially via adaptations within the connective tissue network.

One emerging ECM-targeted approach is the use of hyaluronidase to reduce GAG accumulation by breaking down HA. Imaging studies using T1ρ MRI have demonstrated an increased GAG content in post-stroke spastic muscles; these GAG levels are reduced after hyaluronidase injection (Menon et al., 2019). In addition, a case series on human recombinant hyaluronidase injections reported significant improvements in both passive and active movement, as well as reduced hypertonia severity measured by the MAS, without inducing muscle weakness (Raghavan et al., 2016). While these findings are encouraging, the current evidence base is limited, primarily consisting of uncontrolled case series investigating the treatment effects of hyaluronidase in hypertonic muscle. This level of evidence is insufficient to confirm the efficacy and clinical applicability. Therefore, further clinical trials are needed to evaluate the safety, efficacy, and mechanisms of ECM-targeted therapies in the management of muscle hypertonia.

Overall, our review supports the view that both neural and IMCT/ECM-related factors contribute to muscle hypertonia. Consequently, multimodal treatment strategies that combine neural-targeted interventions with therapies aimed at modifying ECM properties may offer the potential benefit.

### 4.2 Limitations and gaps in the research

This scoping review has some limitations. First, our search was limited to peer-reviewed articles published in English and did not include gray literature or trial registries. As a result, there is a possibility of language and publication bias. This limitation should be considered when interpreting the findings of this review.

In addition, the sample sizes in many studies were relatively small, and there is also a lack of data on progressive neurological disorders such as multiple sclerosis and hereditary spastic paraparesis. These limitations reduce the generalizability of the results and increases the risk of bias. Sampling bias also arose from the differences in study approaches between stroke-induced and CP-induced hypertonia. Stroke-induced hypertonia has been primarily investigated using non-invasive techniques, whereas research on CP-induced spasticity has relied more heavily on muscle biopsies obtained from surgical patients with severe spasticity. Furthermore, even within the same methodological category—whether non-invasive imaging or biopsy-based analysis—significant heterogeneity exists across studies in terms of imaging techniques, biochemical assays, and mechanical testing protocols. This heterogeneity limits direct comparisons between findings and reduces the ability to draw definitive conclusions.

Another substantial gap in this research field is that key ECM components remain underexplored. While collagen accumulation has been extensively studied, the role of other ECM elements, such as elastic fibers, remains largely unexamined. Given the critical function of elastic fibers in tissue flexibility and resilience, understanding their potential alterations in hypertonic muscles is essential.

Lastly, there is a lack of evidence regarding the acute stage of neurological conditions. Most of the included studies focused on chronic populations (such as patients >6 months post-stroke) and individuals with severe hypertonia (such as CP cases requiring surgical intervention). Consequently, little is known about ECM alterations during the early phases of hypertonia. It should be noted that most of the included studies were based on children with CP undergoing surgery for hypertonia, while only a minority investigated stroke-induced hypertonia or SCI-induced hypertonia in adults. This population imbalance limits the generalizability of findings to other patient groups with hypertonia induced by central nervous system disorders. It also increases the risk of bias in the selection of participants, contributing to a high risk of bias rating by ROBINS-E. Additionally, the absence of longitudinal studies makes it difficult to determine when these ECM alterations begin and whether ECM changes are progressive, adaptive, or reversible following therapeutic intervention.

Future research should prioritize standardized assessment methods, include participants in the acute stages of neurological disorders, and conduct longitudinal studies to track ECM remodeling over time. Additionally, efforts should be directed toward developing non-invasive assessment techniques for human studies or establishing animal models to investigate ECM alterations across different conditions. Quantitative, non-invasive techniques such as tensiomyography (Čular et al., 2023), myotonometry (Garcia-Bernal et al., 2021; Lettner et al., 2024), and shear wave elastography (SWE) (Lee et al., 2021; Zúñiga et al., 2021) are already available to evaluate *in vivo* muscle stiffness in both healthy and neurological disorder populations. These tools may serve as valuable additions in future studies investigating IMCT-related muscle stiffness. Such approaches would enable more comprehensive and comparable evaluations of muscle hypertonia, ultimately improving the understanding and treatment of ECM-related stiffness in hypertonic muscles.

## 5 Conclusion

This scoping review provides evidence that alterations in the IMCT composition occur in muscle hypertonia across different conditions, particularly in pediatric CP populations. The alterations of ECM components and their associations with muscle stiffness and spasticity severity demonstrate the effects of ECM remodeling in hypertonia. Targeting these alterations may offer new therapeutic strategies for managing muscle stiffness and improving functional outcomes in patients with hypertonia.

## Data Availability

The original contributions presented in the study are included in the article/Supplementary Material, further inquiries can be directed to the corresponding author.

## References

[B1] Baude M. Nielsen J. B. Gracies J. M. (2019). The neurophysiology of deforming spastic paresis: a revised taxonomy. Ann. Phys. Rehabil. Med. 62 (6), 426–430. 10.1016/j.rehab.2018.10.004 30500361

[B2] Binder-Markey B. I. Broda N. M. Lieber R. L. (2020). Intramuscular anatomy drives collagen content variation within and between muscles. Front. Physiol. 11, 293. 10.3389/fphys.2020.00293 32362834 PMC7181957

[B3] Booth C. M. Cortina-Borja M. J. Theologis T. N. (2001). Collagen accumulation in muscles of children with cerebral palsy and correlation with severity of spasticity. Dev. Med. Child. Neurol. 43 (5), 314–320. 10.1017/s0012162201000597 11368484

[B4] Bowling A. Ebrahim S. (2005). Handbook of health research methods: investigation, measurement and analysis. Education (UK): McGraw-Hill.

[B5] Burke D. Wissel J. Donnan G. A. (2013). Pathophysiology of spasticity in stroke. Neurology 80 (3 Suppl. 2), S20–S26. 10.1212/WNL.0b013e31827624a7 23319482

[B6] Choi J. Do Y. Lee H. (2024). Ultrasound imaging comparison of crural fascia thickness and muscle stiffness in stroke patients with spasticity. Diagn. (Basel) 14 (22), 2606. 10.3390/diagnostics14222606 39594272 PMC11592608

[B7] Contreras O. Rossi F. M. V. Theret M. (2021). Origins, potency, and heterogeneity of skeletal muscle fibro-adipogenic progenitors—time for new definitions. Skelet. Muscle 11 (1), 16. 10.1186/s13395-021-00265-6 34210364 PMC8247239

[B8] Čular D. Babić M. Zubac D. Kezić A. Macan I. Peyré-Tartaruga L. A. (2023). Tensiomyography: from muscle assessment to talent identification tool. Front. Physiol. 14, 1163078. 10.3389/fphys.2023.1163078 37435303 PMC10330706

[B9] Dalise S. Azzollini V. Chisari C. (2020). Brain and muscle: how central nervous system disorders can modify the skeletal muscle. Diagn. (Basel) 10 (12), 1047. 10.3390/diagnostics10121047 33291835 PMC7762031

[B10] de Bruin M. Smeulders M. J. Kreulen M. Huijing P. A. Jaspers R. T. (2014). Intramuscular connective tissue differences in spastic and control muscle: a mechanical and histological study. PLoS One 9 (6), e101038. 10.1371/journal.pone.0101038 24977410 PMC4076209

[B11] Dietz V. Sinkjaer T. (2007). Spastic movement disorder: impaired reflex function and altered muscle mechanics. Lancet Neurol. 6 (8), 725–733. 10.1016/S1474-4422(07)70193-X 17638613

[B12] Evans S. H. Cameron M. W. Burton J. M. (2017). Hypertonia. Curr. Problems Pediatr. Adolesc. Health Care 47 (7), 161–166. 10.1016/j.cppeds.2017.06.005 28716516

[B13] Fede C. Pirri C. Fan C. Petrelli L. Guidolin D. De Caro R. (2021). A closer look at the cellular and molecular components of the deep/Muscular fasciae. Int. J. Mol. Sci. 22 (3), 1411. 10.3390/ijms22031411 33573365 PMC7866861

[B14] Gagliano N. Pelillo F. Chiriva-Internati M. Picciolini O. Costa F. Schutt R. C. (2009). Expression profiling of genes involved in collagen turnover in tendons from cerebral palsy patients. Connect. Tissue Res. 50 (3), 203–208. 10.1080/03008200802613630 19444761

[B15] Gagliano N. Menon A. Martinelli C. Pettinari L. Panou A. Milzani A. (2013). Tendon structure and extracellular matrix components are affected by spasticity in cerebral palsy patients. Muscles Ligaments Tendons J. 3 (1), 42–50. 10.11138/mltj/2013.3.1.042 23885344 PMC3676163

[B16] Ganguly J. Kulshreshtha D. Almotiri M. Jog M. (2021). Muscle Tone physiology and abnormalities. Toxins 13, 282. 10.3390/toxins13040282 33923397 PMC8071570

[B17] Garcia-Bernal M. I. Heredia-Rizo A. M. Gonzalez-Garcia P. Cortés-Vega M. D. Casuso-Holgado M. J. (2021). Validity and reliability of myotonometry for assessing muscle viscoelastic properties in patients with stroke: a systematic review and meta-analysis. Sci. Rep. 11 (1), 5062. 10.1038/s41598-021-84656-1 33658623 PMC7930253

[B18] Gillies A. R. Lieber R. L. (2011). Structure and function of the skeletal muscle extracellular matrix. Muscle Nerve 44 (3), 318–331. 10.1002/mus.22094 21949456 PMC3177172

[B19] Gracies J. M. (2005a). Pathophysiology of spastic paresis. I: paresis and soft tissue changes. Muscle Nerve 31 (5), 535–551. 10.1002/mus.20284 15714510

[B20] Gracies J. M. (2005b). Pathophysiology of spastic paresis. II: emergence of muscle overactivity. Muscle Nerve 31 (5), 552–571. 10.1002/mus.20285 15714511

[B21] Gracies J. M. Alter K. E. Biering-Sørensen B. Dewald J. P. A. Dressler D. Esquenazi A. (2025). Spastic paresis: a treatable movement disorder. Mov. Disord. 40 (1), 44–50. 10.1002/mds.30038 39548808 PMC11752976

[B22] Handsfield G. G. Williams S. Khuu S. Lichtwark G. Stott N. S. (2022). Muscle architecture, growth, and biological remodelling in cerebral palsy: a narrative review. BMC Musculoskelet. Disord. 23 (1), 233. 10.1186/s12891-022-05110-5 35272643 PMC8908685

[B23] Harvey L. A. Katalinic O. M. Herbert R. D. Moseley A. M. Lannin N. A. Schurr K. (2017). Stretch for the treatment and prevention of contracture: an abridged republication of a cochrane systematic review. J. Physiother. 63 (2), 67–75. 10.1016/j.jphys.2017.02.014 28433236

[B24] Higgins J. P. T. Morgan R. L. Rooney A. A. Taylor K. W. Thayer K. A. Silva R. A. (2024). A tool to assess risk of bias in non-randomized follow-up studies of exposure effects (ROBINS-E). Environ. Int. 186, 108602. 10.1016/j.envint.2024.108602 38555664 PMC11098530

[B25] Kielty C. M. Sherratt M. J. Shuttleworth C. A. (2002). Elastic fibres. J. Cell Sci. 115 (14), 2817–2828. 10.1242/jcs.115.14.2817 12082143

[B26] Langevin H. M. Cornbrooks C. J. Taatjes D. J. (2004). Fibroblasts form a body-wide cellular network. Histochem. Cell Biology 122, 7–15. 10.1007/s00418-004-0667-z 15221410

[B27] Lecharte T. Gross R. Nordez A. Le Sant G. (2020). Effect of chronic stretching interventions on the mechanical properties of muscles in patients with stroke: a systematic review. Ann. Phys. Rehabil. Med. 63 (3), 222–229. 10.1016/j.rehab.2019.12.003 31981838

[B28] Lee Y. Kim M. Lee H. (2021). The measurement of stiffness for major muscles with shear wave elastography and myoton: a quantitative analysis study. Diagn. (Basel) 11 (3), 524. 10.3390/diagnostics11030524 33804273 PMC7999852

[B29] Lettner J. Królikowska A. Ramadanov N. Oleksy Ł. Hakam H. T. Becker R. (2024). Evaluating the reliability of MyotonPro in assessing muscle properties: a systematic review of diagnostic test accuracy. Med. Kaunas. 60 (6), 851. 10.3390/medicina60060851 38929468 PMC11205912

[B30] Levac D. Colquhoun H. O'Brien K. K. (2010). Scoping studies: advancing the methodology. Implement. Sci. 5 (1), 69. 10.1186/1748-5908-5-69 20854677 PMC2954944

[B31] Lieber R. L. Steinman S. Barash I. A. Chambers H. (2004). Structural and functional changes in spastic skeletal muscle. Muscle Nerve 29 (5), 615–627. 10.1002/mus.20059 15116365

[B32] Lorentzen J. Pradines M. Gracies J. M. Bo Nielsen J. (2018). On denny-brown’s ‘spastic dystonia’ – what is it and what causes it? Clin. Neurophysiol. 129 (1), 89–94. 10.1016/j.clinph.2017.10.023 29161622

[B33] Malaiya R. McNee A. E. Fry N. R. Eve L. C. Gough M. Shortland A. P. (2007). The morphology of the medial gastrocnemius in typically developing children and children with spastic hemiplegic cerebral palsy. J. Electromyogr. Kinesiol 17 (6), 657–663. 10.1016/j.jelekin.2007.02.009 17459729

[B34] Menon R. G. Raghavan P. Regatte R. R. (2019). Quantifying muscle glycosaminoglycan levels in patients with post-stroke muscle stiffness using T(1ρ) MRI. Sci. Rep. 9 (1), 14513. 10.1038/s41598-019-50715-x 31601831 PMC6787087

[B35] Moretti L. Stalfort J. Barker T. H. Abebayehu D. (2022). The interplay of fibroblasts, the extracellular matrix, and inflammation in scar formation. J. Biol. Chem. 298 (2), 101530. 10.1016/j.jbc.2021.101530 34953859 PMC8784641

[B36] Muiznieks L. D. Keeley F. W. (2013). Molecular assembly and mechanical properties of the extracellular matrix: a fibrous protein perspective. Biochimica Biophysica Acta (BBA) - Mol. Basis Dis. 1832 (7), 866–875. 10.1016/j.bbadis.2012.11.022 23220448

[B37] Noguchi K. S. Mcleod J. C. Phillips S. M. Richardson J. Tang A. (2023). Differences in skeletal muscle fiber characteristics between affected and nonaffected limbs in individuals with stroke: a scoping review. Phys. Ther. 103 (12), pzad095. 10.1093/ptj/pzad095 37478464

[B38] Olsson M. C. Krüger M. Meyer L. H. Ahnlund L. Gransberg L. Linke W. A. (2006). Fibre type-specific increase in passive muscle tension in spinal cord-injured subjects with spasticity. J. Physiol. 577 (Pt 1), 339–352. 10.1113/jphysiol.2006.116749 16931550 PMC2000690

[B39] Purslow P. P. (2020). The structure and role of intramuscular connective tissue in muscle function. Front. Physiol. 11, 495. 10.3389/fphys.2020.00495 32508678 PMC7248366

[B40] Raghavan P. (2018). Emerging therapies for spastic movement disorders. Phys. Med. Rehabil. Clin. N. Am. 29 (3), 633–644. 10.1016/j.pmr.2018.04.004 30626519 PMC6333099

[B41] Raghavan P. Lu Y. Mirchandani M. Stecco A. (2016). Human recombinant hyaluronidase injections for upper limb muscle stiffness in individuals with cerebral injury: a case series. EBioMedicine 9, 306–313. 10.1016/j.ebiom.2016.05.014 27333050 PMC4972484

[B42] Ricard-Blum S. (2011). The collagen family. Cold Spring Harb. Perspect. Biol. 3 (1), a004978. 10.1101/cshperspect.a004978 21421911 PMC3003457

[B43] Ritty T. M. Broekelmann T. J. Werneck C. C. Mecham R. P. (2003). Fibrillin-1 and -2 contain heparin-binding sites important for matrix deposition and that support cell attachment. Biochem. J. 375 (Pt 2), 425–432. 10.1042/BJ20030649 12837131 PMC1223679

[B44] Sherratt M. J. (2009). Tissue elasticity and the ageing elastic fibre, 1574–4647. (Electronic)). 10.1007/s11357-009-9103-6 PMC2813052 19588272

[B45] Smith L. R. Lee K. S. Ward S. R. Chambers H. G. Lieber R. L. (2011). Hamstring contractures in children with spastic cerebral palsy result from a stiffer extracellular matrix and increased *in vivo* sarcomere length. J. Physiol. 589 (10), 2625–2639. 10.1113/jphysiol.2010.203364 21486759 PMC3115830

[B46] Smith L. R. Pichika R. Meza R. C. Gillies A. R. Baliki M. N. Chambers H. G. (2021). Contribution of extracellular matrix components to the stiffness of skeletal muscle contractures in patients with cerebral palsy. Connect. Tissue Research 62 (3), 287–298. 10.1080/03008207.2019.1694011 31779492 PMC7253322

[B47] Stecco A. Stecco C. Raghavan P. (2014). Peripheral mechanisms contributing to spasticity and implications for treatment. Curr. Phys. Med. Rehabilitation Rep. 2, 121–127. 10.1007/s40141-014-0052-3

[B48] Stecco C. Pratt R. Nemetz L. D. Schleip R. Stecco A. Theise N. D. (2025). Towards a comprehensive definition of the human fascial system. J. Anat. 246, 1084–1098. 10.1111/joa.14212 39814456 PMC12079755

[B49] Tang V. W. (2020). Collagen, stiffness, and adhesion: the evolutionary basis of vertebrate mechanobiology. Mol. Biol. Cell 31 (17), 1823–1834. 10.1091/mbc.E19-12-0709 32730166 PMC7525820

[B50] Tisha A. L. Armstrong A. A. Wagoner Johnson A. Lopez-Ortiz C. (2019). Skeletal muscle adaptations and passive muscle stiffness in cerebral palsy: a literature review and conceptual model. J. Appl. Biomech. 35 (1), 68–79. 10.1123/jab.2018-0049 30207207

[B51] Tricco A. C. Lillie E. Zarin W. O'Brien K. K. Colquhoun H. Levac D. (2018). PRISMA extension for scoping reviews (PRISMA-ScR): checklist and explanation. Ann. Intern. Med. 169 (7), 467–473. 10.7326/M18-0850 30178033

[B52] Van den Noort J. C. Bar-On L. Aertbeliën E. Bonikowski M. Braendvik S. M. Broström E. W. (2017). European consensus on the concepts and measurement of the pathophysiological neuromuscular responses to passive muscle stretch. Eur. J. Neurol. 24 (7). 10.1111/ene.13322 28557247

[B53] Von Walden F. Gantelius S. Liu C. Borgström H. Björk L. Gremark O. (2018). Muscle contractures in patients with cerebral palsy and acquired brain injury are associated with extracellular matrix expansion, pro-inflammatory gene expression, and reduced rRNA synthesis. Muscle & Nerve 58 (2), 277–285. 10.1002/mus.26130 29572878

[B54] Wang R. Herman P. Ekeberg Ö. Gäverth J. Fagergren A. Forssberg H. (2017). Neural and non-neural related properties in the spastic wrist flexors: an optimization study. Med. Eng. Phys. 47, 198–209. 10.1016/j.medengphy.2017.06.023 28694106

[B55] Wohlgemuth R. P. Kulkarni V. A. Villalba M. Davids J. R. Smith L. R. (2024). Collagen architecture and biomechanics of gracilis and adductor longus muscles from children with cerebral palsy. J. PHYSIOLOGY-LONDON 602 (14), 3489–3504. 10.1113/JP285988 39008710 PMC11849552

[B56] Yucesoy C. A. Huijing P. A. (2007). Substantial effects of epimuscular myofascial force transmission on muscular mechanics have major implications on spastic muscle and remedial surgery. J. Electromyogr. Kinesiol 17 (6), 664–679. 10.1016/j.jelekin.2007.02.008 17395489

[B57] Zhang W. Liu Y. Zhang H. (2021). Extracellular matrix: an important regulator of cell functions and skeletal muscle development. Cell Biosci. 11 (1), 65. 10.1186/s13578-021-00579-4 33789727 PMC8011170

[B58] Zúñiga L. D. O. López C. A. G. González E. R. (2021). Ultrasound elastography in the assessment of the stiffness of spastic muscles: a systematic review. Ultrasound Med. Biol. 47 (6), 1448–1464. 10.1016/j.ultrasmedbio.2021.01.031 33707090

