# Supplementary material for: Alterations in intramuscular connective tissue in hypertonic muscle: a scoping review
Source: Front Physiol. 2026 Jan 7;16:1720927. doi: 10.3389/fphys.2025.1720927 (PMC12819202; doi:10.3389/fphys.2025.1720927)
Supplement: Supplementary file 1 [file Table1.docx]

Supplementary materials

The alterations of intramuscular connective tissue in hypertonic muscle: A scoping review

Xiaoxiao Zhao, Yunfeng Sun, Caterina Fede, Carmelo Pirri, Wei Gong, Alessandra Del Felice, Carla Stecco

S1. Eligibility form: Inclusion and exclusion criteria

| Factors | Assessment | Comments |
| --- | --- | --- |
| **Article characteristics** |  |  |
| 1. Did the study undergo a full peer review? | YES NO UNCLEAR | If NO /exclude |
| 2. Is it written in English? | YES NO UNCLEAR | If NO /exclude |
| Participants |  |  |
| 3. Were participants diagnosed with neurological disorders with hypertonia | YES NO UNCLEAR | If NO /exclude |
| Methodology |  |  |
| 4. Dose the study tested the structural, biochemical, and historical alteration in IMCT component in hypertonia muscle | YES NO UNCLEAR | If NO /exclude |
| 5. Dose the study focused solely on neural parameters or other non-IMCT parameter (e.g. muscle fiber CSA, fiber type, MyHC isoform, titin isform) alterations in hypertonia |  | If YES /exclude |
| **Outcomes** |  |  |
| 6. did the study reported the IMCT component outcomes | YES NO UNCLEAR | If NO /exclude |
| **FINAL DECISION** | YES NO |  |
| REASONS FOR EXCLUSION FROM REVIEW | | |
| Article type | No full-length article /Review/No English language | |
| Methods | Participants without hypertonia and neurological disorders | |
|  | the study did not evaluate IMCT components alterations to hypertonia | |
| Outcomes | the study did not report IMCT components outcomes of hypertonia | |
| None | Included | |

S2: Search syntax development process.

The review specifically investigates alterations in intramuscular connective tissue (IMCT) in hypertonic muscles.

Accordingly, our search terms were developed around two core domains:

(1) IMCT components (extracellular matrix and cells), and

(2) descriptors of hypertonia.

The details of the search terms and the final search string were constructed.

Search term:

| concepts | synonyms | search terms |
| --- | --- | --- |
| Intramuscular connective tissue |  |  |
| Intramuscular connective tissue | fascia | fascia |
|  | Intramuscular connective tissue | intramuscular connective tissue |
| extracellular matrix components | Extracellular matrix | extracellular matrix |
|  | Elastic fiber/ fibre | (elastic fiber) OR (elastic fibre) |
|  | Collagen fiber/ fibre / collagen type I, II, III, V, IX, X, XVIII, XIII, XI, VII,VI,IV, XII | collagen |
|  | hyaluronan/ hyaluronic acid | hyaluron* |
| Cells components | telocytes | telocytes |
|  | immune cells | immune cells |
|  | myofibroblasts | myofibroblasts |
|  | fibroblasts |  |
| Muscle hypertonia | muscle hypertonia | muscle hypertonia |
|  | muscle spasticity | muscle spasticity |
|  | muscle spastic dystonia | muscle spastic dystonia |
|  | muscle rigidity | muscle rigidity |
|  | muscle hyperreflexia | muscle hyperreflexia |
|  | increased pyramidal tone | increased pyramidal tone |

The final search string is:

((fascia) OR (intramuscular connective tissue) OR (extracellular matrix) OR (elastic fiber) OR (elastic fibre) OR (collagen) OR (hyaluron*) OR (telocytes) OR (immune cells) OR (myofibroblasts) OR (fibroblasts)) AND ((muscle hypertonia) OR (muscle spasticity) OR (muscle spastic dystonia) OR (muscle rigidity) OR (muscle hyperreflexia) OR (increased pyramidal tone))

S3. Literature search strategy on PubMed/Medline

| Database name | PubMed/Medline |
| --- | --- |
| Date searched | 20 July 2025 |
| Search strings | ((fascia) OR (intramuscular connective tissue) OR (extracellular matrix) OR (elastic fiber) OR (elastic fibre) OR (collagen) OR (hyaluron*) OR (telocytes) OR (immune cells) OR (myofibroblasts) OR (fibroblasts)) AND ((muscle hypertonia) OR (muscle spasticity) OR (muscle spastic dystonia) OR (muscle rigidity) OR (muscle hyperreflexia) OR (increased pyramidal tone)). |
| Number of result | 477 |

S4. Literature search strategy on CINAHL

| Database name | CINAHL |
| --- | --- |
| Date searched | 20 July 2025 |
| Search strings | ((fascia) OR (intramuscular connective tissue) OR (extracellular matrix) OR (elastic fiber) OR (elastic fibre) OR (collagen) OR (hyaluron*) OR (telocytes) OR (immune cells) OR (myofibroblasts) OR (fibroblasts)) AND ((muscle hypertonia) OR (muscle spasticity) OR (muscle spastic dystonia) OR (muscle rigidity) OR (muscle hyperreflexia) OR (increased pyramidal tone)). |
| Number of results | 113 |

S5. Literature search strategy on Web of science

| Database name | Web of science |
| --- | --- |
| Date searched | 20 July 2025 |
| Search strings | ((fascia) OR (intramuscular connective tissue) OR (extracellular matrix) OR (elastic fiber) OR (elastic fibre) OR (collagen) OR (hyaluron*) OR (telocytes) OR (immune cells) OR (myofibroblasts) OR (fibroblasts)) AND ((muscle hypertonia) OR (muscle spasticity) OR (muscle spastic dystonia) OR (muscle rigidity) OR (muscle hyperreflexia) OR (increased pyramidal tone)). |
| Number of results | 435 |

S6. Literature search strategy on Scoup

| Database name | Scopus |
| --- | --- |
| Date searched | 20 July 2025 |
| Search strings | ((fascia) OR (intramuscular connective tissue) OR (extracellular matrix) OR (elastic fiber) OR (elastic fibre) OR (collagen) OR (hyaluron*) OR (telocytes) OR (immune cells) OR (myofibroblasts) OR (fibroblasts)) AND ((muscle hypertonia) OR (muscle spasticity) OR (muscle spastic dystonia) OR (muscle rigidity) OR (muscle hyperreflexia) OR (increased pyramidal tone)). |
| Number of results | 308 |

S7. Included studies.

| No. | studies | title | Researcher  1 | Researcher  2 | Finial  decision |
| --- | --- | --- | --- | --- | --- |
| 1 | Booth et al. (2001) [1] | *Collagen accumulation in muscles of children with cerebral palsy and correlation with severity of spasticity* | Y | Y | Y |
| 2 | Malaiya et al. (2007) [2] | *The morphology of the medial gastrocnemius in typically developing children and children with spastic hemiplegic cerebral palsy* | Y | N | Y |
| 3 | Smith et al. (2011)[3] | *Hamstring contractures in children with spastic cerebral palsy result from a stiffer extracellular matrix and increased in vivo sarcomere length* | Y | Y | Y |
| 4 | de Bruin et al. (2014) [4] | *Intramuscular connective tissue differences in spastic and control muscle: a mechanical and histological study* | Y | Y | Y |
| 5 | Smith et al. (2021)[5] | *Contribution of extracellular matrix components to the stiffness of skeletal muscle contractures in patients with cerebral palsy (hand search)* | Y | Y | Y |
| 6 | Gagliano et al. (2013)[6] | *Tendon structure and extracellular matrix components are affected by spasticity in cerebral palsy patients (Hand search)* | Y | Y | Y |
| 7 | Menon et al.(2019) [7] | *Quantifying muscle glycosaminoglycan levels in patients with post-stroke muscle stiffness using T(1ρ) MRI. (hand search)* | Y | Y | Y |
| 8 | Choi et al. (2024)[8] | *Ultrasound Imaging Comparison of Crural Fascia Thickness and Muscle Stiffness in Stroke Patients with Spasticity* | Y | Y | Y |
| 9 | Olsson et al.(2006) [9] | *Fibre type-specific increase in passive muscle tension in spinal cord-injured subjects with spasticity* | y | y | y |
| 10 | Gagliano et al. (2009)  [10] | *Expression Profiling of Genes Involved in Collagen Turnover in Tendons from Cerebral Palsy Patients* | Y | Y | Y |
| 11 | Galvão et al. (2022)  [11] | *Shear Wave Elastography of the Brachioradialis Spastic Muscle and Its Correlations with Biceps Brachialis and Clinical Scales’. Clinical Biomechanics* | ? | ? | N |
| 12 | Leonard et al. (2019)  [12] | Stiffness of hip adductor myofibrils is decreased in children with spastic cerebral palsy | N | Y | N |
| 13 | Chardon et al. (2020)  [13] | In-Vivo Study of Passive Musculotendon Mechanics in Chronic Hemispheric Stroke Survivors | ? | ? | N |
| 14 | Wohlgemuth et al. (2024)[14] | Collagen architecture and biomechanics of gracilis and adductor longus muscles from children with cerebral palsy | Y | Y | Y |
| 15 | Güvener et al. (2021) [15] | Are COL4A1 and COL4A2 gene polymorphisms associated with cerebral palsy? | N | N | N |
| 16 | Von Walden et al. (2018)[16] | Muscle contractures in patients with cerebral palsy and acquired brain injury are associated with extracellular matrix expansion, pro-inflammatory gene expression, and reduced rRNA synthesis | Y | Y | Y |

S8. Inter-rater reliability calculation[17].

|  |  | Reviewer (YS) | | |  |
| --- | --- | --- | --- | --- | --- |
|  |  | Include | Exclude | Unsure | Total |
| Reviewer (XZ) | Include | 11 | 1 | 0 | 12 |
|  | Exclude | 1 | 1 | 0 | 2 |
|  | Unsure | 0 | 0 | 2 | 2 |
|  | Total | 12 | 2 | 2 | 16 |

PO = (11+1+2)/16 = 15/16=0.9375

PE = (12*12+ 2*2 + 2*2)/(16*16) = (144+4+4)/256= 152/256=0.59375

Kappa = (PO - PE)/(1- PE) = (0.9375-0.59375)/(1-0.59375)=. 0.34375/0.40625=0. 846

S9. Studies that might appear to meet the inclusion criteria, but excluded after full-text articles review and discussion (n=4)

| **Studies** | **title** | **Reason for Exclusion** |
| --- | --- | --- |
| Galvão et al. (2022) | Shear Wave Elastography of the Brachioradialis Spastic Muscle and Its Correlations with Biceps Brachialis and Clinical Scales’. Clinical Biomechanics | the study did not evaluate and report IMCT outcomes of hypertonia. |
| Chardon et al. (2020) | In-Vivo Study of Passive Musculotendon Mechanics in Chronic Hemispheric Stroke Survivors | the study did not evaluate and report IMCT outcomes of hypertonia. |
| Leonard et al. (2019) | Stiffness of hip adductor myofibrils is decreased in children with spastic cerebral palsy | the study did not evaluate and report IMCT parameters outcomes of hypertonia |
| Güvener et al. (2021) | Are COL4A1 and COL4A2 gene polymorphisms associated with cerebral palsy? | the study did not evaluate and report IMCT parameters outcomes of hypertonia |

S10. Risk of bias of the studies

| **Studies** | **D1** | **D2** | **D3** | **D4** | **D5** | **D6** | **D7** | **overall** |
| --- | --- | --- | --- | --- | --- | --- | --- | --- |
| Booth et al. (2001) | Low | Low | High | Low | Low | Low | Low | High |
| Malaiya et al. (2007) | Low | Low | High | Low | Low | Low | Low | High |
| Gagliano et al. (2009) | Some concern | Low | High | Low | Low | Low | Low | High |
| Smith et al. (2011) | Low | Low | High | Low | Low | Low | Low | High |
| de Bruin et al. (2014) | Some concern | Low | High | Low | Low | Low | Low | High |
| Smith et al. (2021) | Some concern | Low | High | Low | Low | Low | Low | High |
| Gagliano et al. (2013) | Some concern | Low | High | Low | Low | Low | Low | High |
| Menon et al. (2019) | Some concern | Low | High | Low | Low | Low | Low | High |
| Choi et al. (2024) | Some concern | Low | High | Low | Low | Low | Low | High |
| Olsson et al.(2006) | Some concern | Low | High | Low | Low | Low | Low | High |
| Wohlgemuth et al. (2024) | Some concern | low | High | Low | Low | Low | Low | High |
| Von Walden et al. (2018) | Some concern | low | High | Low | Low | Low | Low | High |

1. Booth, C. M.; Cortina-Borja, M. J.; Theologis, T. N., Collagen accumulation in muscles of children with cerebral palsy and correlation with severity of spasticity. *Dev Med Child Neurol* **2001,** 43, (5), 314-20.

2. Malaiya, R.; McNee, A. E.; Fry, N. R.; Eve, L. C.; Gough, M.; Shortland, A. P., The morphology of the medial gastrocnemius in typically developing children and children with spastic hemiplegic cerebral palsy. *J Electromyogr Kinesiol* **2007,** 17, (6), 657-63.

3. Smith, L. R.; Lee, K. S.; Ward, S. R.; Chambers, H. G.; Lieber, R. L., Hamstring contractures in children with spastic cerebral palsy result from a stiffer extracellular matrix and increased in vivo sarcomere length. *J Physiol* **2011,** 589, (Pt 10), 2625-39.

4. de Bruin, M.; Smeulders, M. J.; Kreulen, M.; Huijing, P. A.; Jaspers, R. T., Intramuscular connective tissue differences in spastic and control muscle: a mechanical and histological study. *PLoS One* **2014,** 9, (6), e101038.

5. Smith, L. R.; Pichika, R.; Meza, R. C.; Gillies, A. R.; Baliki, M. N.; Chambers, H. G.; Lieber, R. L., Contribution of extracellular matrix components to the stiffness of skeletal muscle contractures in patients with cerebral palsy. *Connective tissue research* **2021,** 62, (3), 287-298.

6. Gagliano, N.; Menon, A.; Martinelli, C.; Pettinari, L.; Panou, A.; Milzani, A.; Dalle-Donne, I.; Portinaro, N. M., Tendon structure and extracellular matrix components are affected by spasticity in cerebral palsy patients. *Muscles Ligaments Tendons J* **2013,** 3, (1), 42-50.

7. Menon, R. G.; Raghavan, P.; Regatte, R. R., Quantifying muscle glycosaminoglycan levels in patients with post-stroke muscle stiffness using T(1ρ) MRI. *Sci Rep* **2019,** 9, (1), 14513.

8. Choi, J.; Do, Y.; Lee, H., Ultrasound Imaging Comparison of Crural Fascia Thickness and Muscle Stiffness in Stroke Patients with Spasticity. *Diagnostics (Basel)* **2024,** 14, (22).

9. Olsson, M. C.; Krüger, M.; Meyer, L. H.; Ahnlund, L.; Gransberg, L.; Linke, W. A.; Larsson, L., Fibre type-specific increase in passive muscle tension in spinal cord-injured subjects with spasticity. *J Physiol* **2006,** 577, (Pt 1), 339-52.

10. Gagliano, N.; Pelillo, F.; Chiriva-Internati, M.; Picciolini, O.; Costa, F.; Schutt, R. C., Jr.; Gioia, M.; Portinaro, N., Expression profiling of genes involved in collagen turnover in tendons from cerebral palsy patients. *Connect Tissue Res* **2009,** 50, (3), 203-8.

11. Galvão, S.; de Oliveira, L. F.; de Lima, R.; Xerez, D.; Menegaldo, L. L., Shear wave elastography of the brachioradialis spastic muscle and its correlations with biceps brachialis and clinical scales. *Clin Biomech (Bristol, Avon)* **2022,** 97, 105687.

12. Leonard, T. R.; Howard, J. J.; Larkin-Kaiser, K.; Joumaa, V.; Logan, K.; Orlik, B.; El-Hawary, R.; Gauthier, L.; Herzog, W., Stiffness of hip adductor myofibrils is decreased in children with spastic cerebral palsy. *J Biomech* **2019,** 87, 100-106.

13. Chardon, M. K.; Suresh, N. L.; Dhaher, Y. Y.; Rymer, W. Z., In-Vivo Study of Passive Musculotendon Mechanics in Chronic Hemispheric Stroke Survivors. *IEEE Trans Neural Syst Rehabil Eng* **2020,** 28, (4), 1022-1031.

14. Wohlgemuth, R. P.; Kulkarni, V. A.; Villalba, M.; Davids, J. R.; Smith, L. R., Collagen architecture and biomechanics of gracilis and adductor longus muscles from children with cerebral palsy. *JOURNAL OF PHYSIOLOGY-LONDON* **2024,** 602, (14), 3489-3504.

15. Güvener, O.; Sezgin, M.; Tezol, Ö.; Barlas, İ. Ö.; Özdemir, A. A.; Kanık, E. A., Are COL4A1 and COL4A2 gene polymorphisms associated with cerebral palsy? *Turkish Journal of Physical Medicine & Rehabilitation (2587-1250)* **2021,** 67, (2), 242-249.

16. Von Walden, F.; Gantelius, S.; Liu, C.; Borgström, H.; Björk, L.; Gremark, O.; Stål, P.; Nader, G. A.; PontéN, E., Muscle contractures in patients with cerebral palsy and acquired brain injury are associated with extracellular matrix expansion, pro-inflammatory gene expression, and reduced rRNA synthesis. *MUSCLE & NERVE* **2018,** 58, (2), 277-285.

17. Higgins, J. P. T.; Wells, G. A., Cochrane handbook for systematic reviews of interventions. **2011**.
