# Supplementary material for: Alterations in intramuscular connective tissue in hypertonic muscle: a scoping review
Source: Front Physiol. 2026 Jan 7;16:1720927. doi: 10.3389/fphys.2025.1720927 (PMC12819202; doi:10.3389/fphys.2025.1720927)
Supplement: Supplementary file 2 [file DataSheet1.pdf]

Supplementary materials

The alterations of intramuscular connective tissue in hypertonic muscle: A scoping review

Xiaoxiao Zhao, Yunfeng Sun, Caterina Fede, Carmelo Pirri, Wei Gong, Alessandra Del Felice, Carla Stecco

S1. Eligibility form: Inclusion and exclusion criteria

Factors	Assessment	Comments
**Article characteristics**		
1. Did the study undergo a full peer review?	YES NO UNCLEAR	If NO /exclude
2. Is it written in English?	YES NO UNCLEAR	If NO /exclude
Participants		
3. Were participants diagnosed with neurological disorders with hypertonia	YES NO UNCLEAR	If NO /exclude
Methodology		
4. Dose the study tested the structural, biochemical, and historical alteration in IMCT component in hypertonia muscle	YES NO UNCLEAR	If NO /exclude
5. Dose the study focused solely on neural parameters or other non-IMCT parameter (e.g. muscle fiber CSA, fiber type, MyHC isoform, titin isform) alterations in hypertonia		If YES /exclude
**Outcomes**		
6. did the study reported the IMCT component outcomes	YES NO UNCLEAR	If NO /exclude
**FINAL DECISION**	YES NO	
REASONS FOR EXCLUSION FROM REVIEW		
Article type	No full-length article /Review/No English language	
Methods	Participants without hypertonia and neurological disorders	
	the study did not evaluate IMCT components alterations to hypertonia	
Outcomes	the study did not report IMCT components outcomes of hypertonia	
None	Included	

S2: Search syntax development process.

The review specifically investigates alterations in intramuscular connective tissue (IMCT) in hypertonic muscles.

Accordingly, our search terms were developed around two core domains:

(1) IMCT components (extracellular matrix and cells), and

(2) descriptors of hypertonia.

The details of the search terms and the final search string were constructed.

Search term:

concepts	synonyms	search terms
Intramuscular connective tissue		
Intramuscular connective tissue	fascia	fascia
	Intramuscular connective tissue	intramuscular connective tissue
extracellular matrix components	Extracellular matrix	extracellular matrix
	Elastic fiber/ fibre	(elastic fiber) OR (elastic fibre)
	Collagen fiber/ fibre / collagen type I, II, III, V, IX, X, XVIII, XIII, XI, VII,VI,IV, XII	collagen
	hyaluronan/ hyaluronic acid	hyaluron*
Cells components	telocytes	telocytes
	immune cells	immune cells
	myofibroblasts	myofibroblasts
	fibroblasts	

Muscle hypertonia	muscle hypertonia	muscle hypertonia
	muscle spasticity	muscle spasticity
	muscle spastic dystonia	muscle spastic dystonia
	muscle rigidity	muscle rigidity
	muscle hyperreflexia	muscle hyperreflexia
	increased pyramidal tone	increased pyramidal tone

The final search string is:

((fascia) OR (intramuscular connective tissue) OR (extracellular matrix) OR (elastic fiber) OR (elastic fibre) OR (collagen) OR (hyaluron*) OR (telocytes) OR (immune cells) OR (myofibroblasts) OR (fibroblasts)) AND ((muscle hypertonia) OR (muscle spasticity) OR (muscle spastic dystonia) OR (muscle rigidity) OR (muscle hyperreflexia) OR (increased pyramidal tone))

S3. Literature search strategy on PubMed/Medline

Database name	PubMed/Medline
Date searched	20 July 2025
Search strings	((fascia) OR (intramuscular connective tissue) OR (extracellular matrix) OR (elastic fiber) OR (elastic fibre) OR (collagen) OR (hyaluron*) OR (telocytes) OR (immune cells) OR (myofibroblasts) OR (fibroblasts)) AND ((muscle hypertonia) OR (muscle spasticity) OR (muscle spastic dystonia) OR (muscle rigidity) OR (muscle hyperreflexia) OR (increased pyramidal tone)).
Number of result	477

S4. Literature search strategy on CINAHL

Database name	CINAHL
Date searched	20 July 2025
Search strings	((fascia) OR (intramuscular connective tissue) OR (extracellular matrix) OR (elastic fiber) OR (elastic fibre) OR (collagen) OR (hyaluron*) OR (telocytes) OR (immune cells) OR (myofibroblasts) OR (fibroblasts)) AND ((muscle hypertonia) OR (muscle spasticity) OR (muscle spastic dystonia) OR (muscle rigidity) OR (muscle hyperreflexia) OR (increased pyramidal tone)).
Number of results	113

S5. Literature search strategy on Web of science

Database name	Web of science
Date searched	20 July 2025
Search strings	((fascia) OR (intramuscular connective tissue) OR (extracellular matrix) OR (elastic fiber) OR (elastic fibre) OR (collagen) OR (hyaluron*) OR (telocytes) OR (immune cells) OR (myofibroblasts) OR (fibroblasts)) AND ((muscle hypertonia) OR (muscle spasticity) OR (muscle spastic dystonia) OR (muscle rigidity) OR (muscle hyperreflexia) OR (increased pyramidal tone)).
Number of results	435

S6. Literature search strategy on Scoup

Database name	Scopus
Date searched	20 July 2025
Search strings	((fascia) OR (intramuscular connective tissue) OR (extracellular matrix) OR (elastic fiber) OR (elastic fibre) OR (collagen) OR (hyaluron*) OR (telocytes) OR (immune cells) OR (myofibroblasts) OR (fibroblasts)) AND ((muscle hypertonia) OR (muscle spasticity) OR (muscle spastic dystonia) OR (muscle rigidity) OR (muscle hyperreflexia) OR (increased pyramidal tone)).
Number of results	308

S7. Included studies.

No.	studies	title	Researcher 1	Researcher 2	Finial decision
1	Booth et al. (2001) [1]	*Collagen accumulation in muscles of children with cerebral palsy and correlation with severity of spasticity*	Y	Y	Y
2	Malaiya et al. (2007) [2]	*The morphology of the medial gastrocnemius in typically developing children and children with spastic hemiplegic cerebral palsy*	Y	N	Y
3	Smith et al. (2011)[3]	*Hamstring contractures in children with spastic cerebral palsy result from a stiffer extracellular matrix and increased in vivo sarcomere length*	Y	Y	Y

4	de Bruin et al. (2014) [4]	*Intramuscular connective tissue differences in spastic and control muscle: a mechanical and histological study*	Y	Y	Y
5	Smith et al. (2021)[5]	*Contribution of extracellular matrix components to the stiffness of skeletal muscle contractures in patients with cerebral palsy (hand search)*	Y	Y	Y
6	Gagliano et al. (2013)[6]	*Tendon structure and extracellular matrix components are affected by spasticity in cerebral palsy patients (Hand search)*	Y	Y	Y
7	Menon et al.(2019) [7]	*Quantifying muscle glycosaminoglycan levels in patients with post-stroke muscle stiffness using T(1ρ) MRI. (hand search)*	Y	Y	Y
8	Choi et al. (2024)[8]	*Ultrasound Imaging Comparison of Crural Fascia Thickness and Muscle Stiffness in Stroke Patients with Spasticity*	Y	Y	Y
9	Olsson et al.(2006) [9]	*Fibre type-specific increase in passive muscle tension in spinal cord-injured subjects with spasticity*	y	y	y
10	Gagliano et al. (2009) [10]	*Expression Profiling of Genes Involved in Collagen Turnover in Tendons from Cerebral Palsy Patients*	Y	Y	Y
11	Galvão et al. (2022) [11]	*Shear Wave Elastography of the Brachioradialis Spastic Muscle and Its Correlations with Biceps Brachialis and Clinical Scales'. Clinical Biomechanics*	?	?	N

12	Leonard et al. (2019)<br><br>[12]	Stiffness of hip adductor myofibrils is decreased in children with spastic cerebral palsy	N	Y	N
13	Chardon et al. (2020)<br><br>[13]	In-Vivo Study of Passive Musculotendon Mechanics in Chronic Hemispheric Stroke Survivors	?	?	N
14	Wohlgemuth et al. (2024)[14]	Collagen architecture and biomechanics of gracilis and adductor longus muscles from children with cerebral palsy	Y	Y	Y
15	Güvener et al. (2021) [15]	Are COL4A1 and COL4A2 gene polymorphisms associated with cerebral palsy?	N	N	N
16	Von Walden et al. (2018)[16]	Muscle contractures in patients with cerebral palsy and acquired brain injury are associated with extracellular matrix expansion, pro-inflammatory gene expression, and reduced rRNA synthesis	Y	Y	Y

S8. Inter-rater reliability calculation[17].

		Reviewer (YS)			
		Include	Exclude	Unsure	Total
Reviewer (XZ)	Include	11	1	0	12
	Exclude	1	1	0	2
	Unsure	0	0	2	2
	Total	12	2	2	16

PO = (11+1+2)/16 = 15/16=0.9375

PE = (12*12+ 2*2 + 2*2)/(16*16) = (144+4+4)/256= 152/256=0.59375

Kappa = (PO - PE)/(1- PE) = (0.9375-0.59375)/(1-0.59375)=. 0.34375/0.40625=0. 846

S9. Studies that might appear to meet the inclusion criteria, but excluded after full-text articles review and discussion (n=4)

**Studies**	**title**	**Reason for Exclusion**
Galvão et al. (2022)	Shear Wave Elastography of the Brachioradialis Spastic Muscle and Its Correlations with Biceps Brachialis and Clinical Scales'. Clinical Biomechanics	the study did not evaluate and report IMCT outcomes of hypertonia.
Chardon et al. (2020)	In-Vivo Study of Passive Musculotendon Mechanics in Chronic Hemispheric Stroke Survivors	the study did not evaluate and report IMCT outcomes of hypertonia.
Leonard et al. (2019)	Stiffness of hip adductor myofibrils is decreased in children with spastic cerebral palsy	the study did not evaluate and report IMCT parameters outcomes of hypertonia
Güvener et al. (2021)	Are COL4A1 and COL4A2 gene polymorphisms associated with cerebral palsy?	the study did not evaluate and report IMCT parameters outcomes of hypertonia

S10. Risk of bias of the studies

Studies	D1	D2	D3	D4	D5	D6	D7	overall
Booth et al. (2001)	Low	Low	High	Low	Low	Low	Low	High
Malaiya et al. (2007)	Low	Low	High	Low	Low	Low	Low	High
Gagliano et al. (2009)	Some concern	Low	High	Low	Low	Low	Low	High
Smith et al. (2011)	Low	Low	High	Low	Low	Low	Low	High
de Bruin et al. (2014)	Some concern	Low	High	Low	Low	Low	Low	High
Smith et al. (2021)	Some concern	Low	High	Low	Low	Low	Low	High
Gagliano et al. (2013)	Some concern	Low	High	Low	Low	Low	Low	High
Menon et al. (2019)	Some concern	Low	High	Low	Low	Low	Low	High
Choi et al. (2024)	Some concern	Low	High	Low	Low	Low	Low	High
Olsson et al.(2006)	Some concern	Low	High	Low	Low	Low	Low	High
Wohlgemuth et al. (2024)	Some concern	low	High	Low	Low	Low	Low	High
Von Walden et al. (2018)	Some concern	low	High	Low	Low	Low	Low	High

1. Booth, C. M.; Cortina-Borja, M. J.; Theologis, T. N., Collagen accumulation in muscles of children with cerebral palsy and correlation with severity of spasticity. *Dev Med Child Neurol* **2001,** 43, (5), 314-20.
2. Malaiya, R.; McNee, A. E.; Fry, N. R.; Eve, L. C.; Gough, M.; Shortland, A. P., The morphology of the medial gastrocnemius in typically developing children and children with spastic hemiplegic cerebral palsy. *J Electromyogr Kinesiol* **2007,** 17, (6), 657-63.
3. Smith, L. R.; Lee, K. S.; Ward, S. R.; Chambers, H. G.; Lieber, R. L., Hamstring contractures in children with spastic cerebral palsy result from a stiffer extracellular matrix and increased in vivo sarcomere length. *J Physiol* **2011,** 589, (Pt 10), 2625-39.
4. de Bruin, M.; Smeulders, M. J.; Kreulen, M.; Huijing, P. A.; Jaspers, R. T., Intramuscular connective tissue differences in spastic and control muscle: a mechanical and histological study. *PLoS One* **2014,** 9, (6), e101038.
5. Smith, L. R.; Pichika, R.; Meza, R. C.; Gillies, A. R.; Baliki, M. N.; Chambers, H. G.; Lieber, R. L., Contribution of extracellular matrix components to the stiffness of skeletal muscle contractures in patients with cerebral palsy. *Connective tissue research* **2021,** 62, (3), 287-298.
6. Gagliano, N.; Menon, A.; Martinelli, C.; Pettinari, L.; Panou, A.; Milzani, A.; Dalle-Donne, I.; Portinaro, N. M., Tendon structure and extracellular matrix components are affected by spasticity in cerebral palsy patients. *Muscles Ligaments Tendons J* **2013,** 3, (1), 42-50.
7. Menon, R. G.; Raghavan, P.; Regatte, R. R., Quantifying muscle glycosaminoglycan levels in patients with post-stroke muscle stiffness using T(1ρ) MRI. *Sci Rep* **2019,** 9, (1), 14513.
8. Choi, J.; Do, Y.; Lee, H., Ultrasound Imaging Comparison of Crural Fascia Thickness and Muscle Stiffness in Stroke Patients with Spasticity. *Diagnostics (Basel)* **2024,** 14, (22).
9. Olsson, M. C.; Krüger, M.; Meyer, L. H.; Ahnlund, L.; Gransberg, L.; Linke, W. A.; Larsson, L., Fibre type-specific increase in passive muscle tension in spinal cord-injured subjects with spasticity. *J Physiol* **2006,** 577, (Pt 1), 339-52.
10. Gagliano, N.; Pelillo, F.; Chiriva-Internati, M.; Picciolini, O.; Costa, F.; Schutt, R. C., Jr.; Gioia, M.; Portinaro, N., Expression profiling of genes involved in collagen turnover in tendons from cerebral palsy patients. *Connect Tissue Res* **2009,** 50, (3), 203-8.
11. Galvão, S.; de Oliveira, L. F.; de Lima, R.; Xerez, D.; Menegaldo, L. L., Shear wave elastography of the brachioradialis spastic muscle and its correlations with biceps brachialis and clinical scales. *Clin Biomech (Bristol, Avon)* **2022,** 97, 105687.
12. Leonard, T. R.; Howard, J. J.; Larkin-Kaiser, K.; Joumaa, V.; Logan, K.; Orlik, B.; El-Hawary, R.; Gauthier, L.; Herzog, W., Stiffness of hip adductor myofibrils is decreased in children with spastic cerebral palsy. *J Biomech* **2019,** 87, 100-106.
13. Chardon, M. K.; Suresh, N. L.; Dhaher, Y. Y.; Rymer, W. Z., In-Vivo Study of Passive Musculotendon Mechanics in Chronic Hemispheric Stroke Survivors. *IEEE Trans Neural Syst Rehabil Eng* **2020,** 28, (4), 1022-1031.
14. Wohlgemuth, R. P.; Kulkarni, V. A.; Villalba, M.; Davids, J. R.; Smith, L. R., Collagen architecture and biomechanics of gracilis and adductor longus muscles from children with cerebral palsy. *JOURNAL OF PHYSIOLOGY-LONDON* **2024,** 602, (14), 3489-3504.

15. Güvener, O.; Sezgin, M.; Tezol, Ö.; Barlas, İ. Ö.; Özdemir, A. A.; Kanık, E. A., Are COL4A1 and COL4A2 gene polymorphisms associated with cerebral palsy? *Turkish Journal of Physical Medicine & Rehabilitation (2587-1250)* **2021,** 67, (2), 242-249.
16. Von Walden, F.; Gantelius, S.; Liu, C.; Borgström, H.; Björk, L.; Gremark, O.; Stål, P.; Nader, G. A.; PontéN, E., Muscle contractures in patients with cerebral palsy and acquired brain injury are associated with extracellular matrix expansion, pro-inflammatory gene expression, and reduced rRNA synthesis. *MUSCLE & NERVE* **2018,** 58, (2), 277-285.
17. Higgins, J. P. T.; Wells, G. A., Cochrane handbook for systematic reviews of interventions. **2011**.
